# Sclerosing Pneumocytoma With Pulmonary Sarcoidosis: A Case Report

**DOI:** 10.7759/cureus.31265

**Published:** 2022-11-08

**Authors:** Evan Brydges, Matthew Cecchini, Sherman Lin, Michael Mitchell

**Affiliations:** 1 Internal Medicine, London Health Sciences Centre, London, CAN; 2 Pathology and Laboratory Medicine, London Health Sciences Centre, London, CAN; 3 Pulmonary Medicine, London Health Sciences Centre, London, CAN

**Keywords:** thoracic imaging, lung pathology, pulmonary mass, pulmonary sarcoidosis, pneumocytoma

## Abstract

Pulmonary sclerosing pneumocytoma, formally named pulmonary sclerosing hemangioma, is a rare benign tumor with malignant potential often identified as solid pulmonary nodules. Sarcoidosis is an inflammatory, multisystemic disease of unknown cause with a wide range of clinical manifestations. The disorder is characterized by the formation of noncaseating granulomas in virtually any organ in the body. We present a case of a patient presenting with fever, weight loss, and respiratory symptoms found to have both a sclerosing pneumocytoma and pulmonary sarcoidosis. A diagnosis was made following the lobectomy. The patient was followed for two years with stable lymphadenopathy while remaining asymptomatic.

## Introduction

First described by Liebow in 1956, pulmonary sclerosing pneumocytoma, formally named pulmonary sclerosing hemangioma, is a rare benign tumor with malignant potential [1]. This was first believed to be a variant of hemangioma, but after further immunohistochemical and genetic studies, sclerosing hemangioma is now considered an epithelial tumor of pulmonary origin [2,3]. Pneumocytomas are often identified as a solid pulmonary nodules [4] but have also been shown to metastasize to hilar lymph nodes [5]; however, this has not been shown to affect prognosis [6]. Seen more often in women in their fifth decade, 95% of the cases are peripheral lung lesions [3]. Computerized tomography (CT) findings typically identify a well-defined, round, or ovoid juxta pleural nodule or mass [7]. These nodules typically show low to moderate uptake on FDG PET scans [7,8]. Most patients remain asymptomatic, and the tumor is usually detected incidentally during routine radiological examinations for other reasons [3].

Sarcoidosis is an inflammatory, multisystemic disease of unknown cause with a wide range of clinical manifestations believed to have been first described by Dr. Jonathan Hutchinson in 1877. The formation of noncaseating granulomas characterizes the disorder. It can affect virtually any organ in the body, predominantly the lungs, which is present in over 90% of patients [9,10]. The incidence has been reported to peak at 30 to 50 years of age in men and 50 to 60 in women [11]. Sarcoidosis typically has non-necrotizing granulomatous inflammation on histopathologic examination. These findings are not completely specific, with the diagnosis being made by having a compatible clinical presentation with the exclusion of other causes of granulomatous inflammation [12]. Sarcoid granulomas are most frequently located along the perilymphatic regions and the broncho vascular branches [13]. Here we present the workup and diagnosis of a patient found to have both sclerosing pneumocytoma and pulmonary sarcoidosis.

## Case presentation

**Figure 1:**
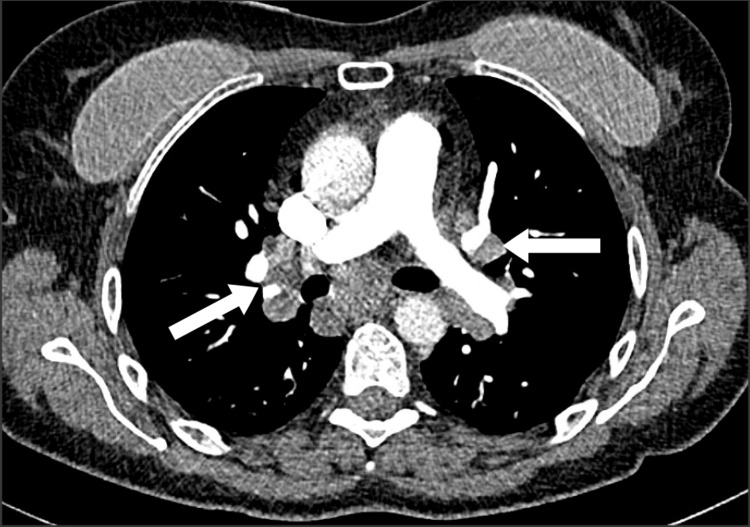
CT scan of mediastinal and bilateral hilar lymphadenopathy

**Figure 2:**
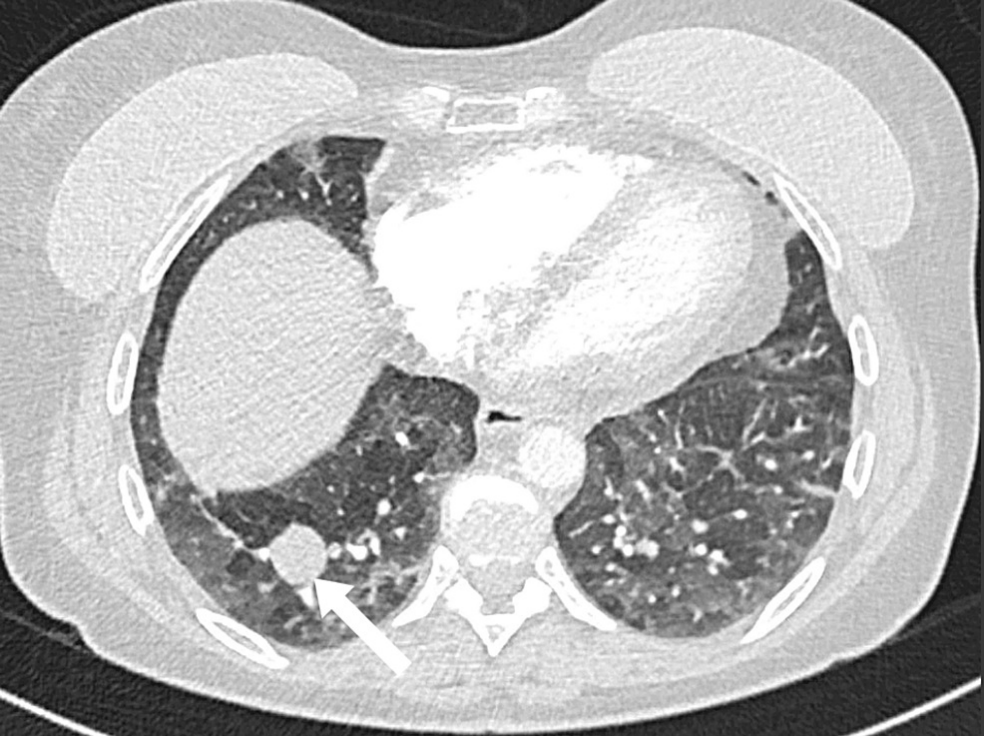
CT scan of right lower lobe nodule (1.9 x 1.9 cm)

**Figure 3:**
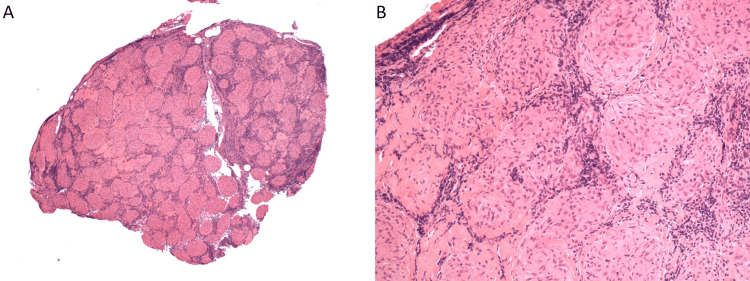
(A) Low power photomicrograph (objective magnification 2x) of right lower paratracheal location 4R with lymph node largely replaced by well-formed non-necrotizing granuloma. (B) Higher power photomicrograph (objective magnification 10x) of granuloma composed of tight collections of epithelioid histocytes.

**Figure 4:**
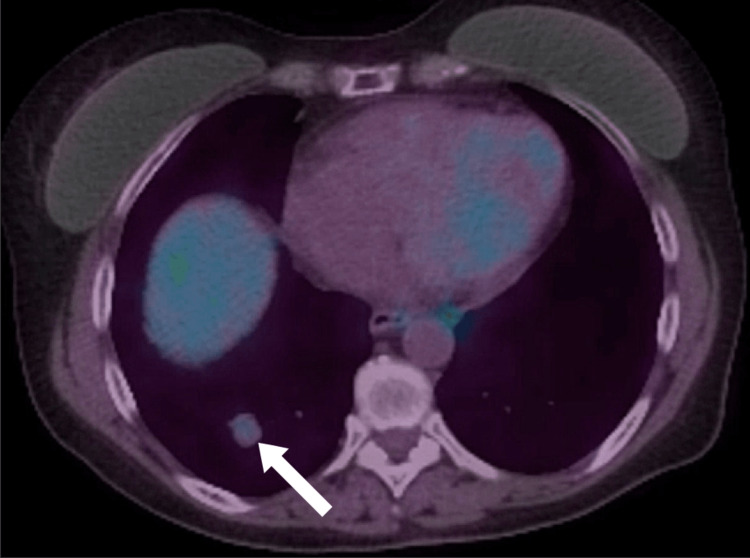
PET scan of right lower lobe lung nodule with trivial FDG-avidity (SUV 1.8)

**Figure 5:**
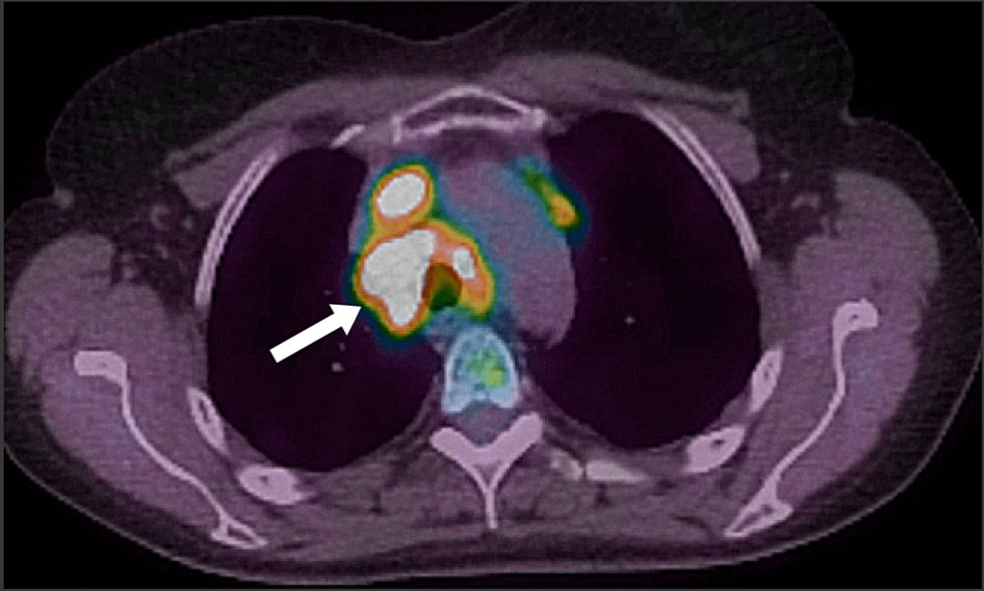
PET scan of mediastinal and hilar lymph nodes with high FDG-avidity (subcarinal lymph node - SUV of 15.7)

A 56-year-old woman presented to the emergency department with fever, 10-pound (4.54 kg) weight loss over three weeks, weakness, shortness of breath, and dry cough. She had a history of depression and was taking no regular medications. She had a four-pack-year history of smoking and quit at age 22. She had no symptoms of joint pain, rashes, uveitis, syncope, or palpitations. An initial chest x-ray was reported as normal. A thorax CT identified a right lower lobe nodule measuring 1.9 x 1.9 cm (Figure 1). There was also noted to be marked mediastinal and bilateral hilar lymphadenopathy (Figure 2). She subsequently underwent pulmonary function testing, which showed normal spirometry but evidence of hyperinflation and gas trapping (FEV1/FVC 87%, FEV1 114% of predicted, FVC 111% of predicted, TLC 127% of predicted, RV 151% of predicted, DLCO 87% of predicted). Due to the concern of an underlying malignancy, an endobronchial ultrasound with transbronchial needle aspiration (EBUS-TBNA) was performed on the paratracheal (station 4R) (Figures 3A, 3B) and subcarinal (station 7) lymph nodes. Cytopathology from these samples showed only a single epithelioid granuloma without evidence of malignancy and was interpreted as non-diagnostic. A subsequent PET scan found the right lower lobe lung nodule had only trivial FDG-avidity (SUV 1.8) (Figure 4). At the same time, the mediastinal and hilar lymph nodes were intensely hypermetabolic (the subcarinal lymph node had an SUV of 15.7) (Figure 5). A CT-guided core-needle biopsy of the right lower lobe lung nodule was performed. The core needle biopsy identified a neoplasm with no nuclear atypia, necrosis, or mitotic activity with diffuse staining for TTF-1 and focal and variable staining for cytokeratins (AE1/AE3, 7, and CAM5.2). The lesional cells were negative for synaptophysin, S100, CD34, desmin, smooth muscle actin, and p40. GMS and PAS-D histochemical stains were negative for fungal organisms. ZN histochemical stain was negative for acid-fast organisms. Given the imaging results and neoplasm identified on biopsy, there remained a significant concern for primary lung adenocarcinoma, but a sclerosing pneumocytoma was also raised in the differential diagnosis based on the limited tissue in the core needle biopsy.

 

**Figure 6:**
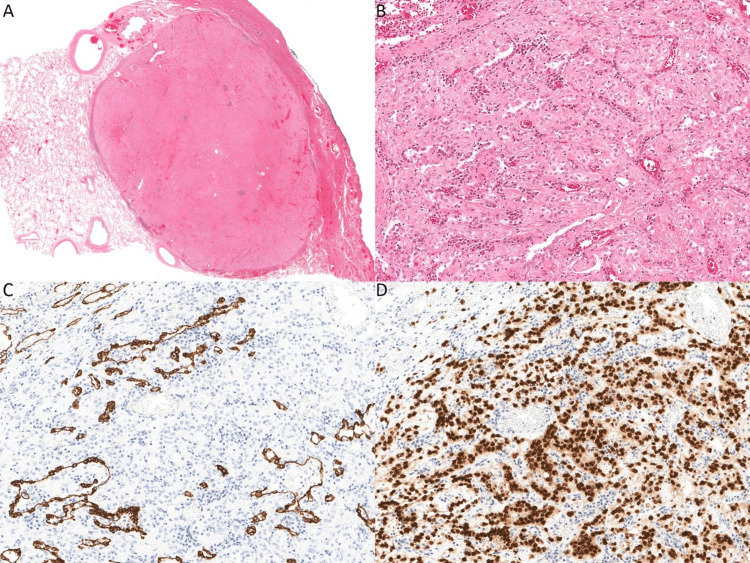
Pathology of resected nodule (A), round stromal cells (B), surface cells positive for cytokeratin 7 and TTF-1 (C), and round stromal cells positive for only TTF-1 (D).

Given the diagnostic limitations of a small tissue sample from the needle biopsy, a definitive diagnosis could not be made; therefore, the patient underwent a right thoracotomy with right lower lobectomy and lymph node resection to allow examination of the entire lesion. On histopathology, a 2.3 cm well-demarcated nodule was identified (Figure 6A). The nodule was composed of cuboidal surface cells and round stromal cells. The round stromal cells had a predominately solid architecture (Figure 6B). The surface cuboidal cells were morphologically similar to type II pneumocytes, and the round cells had bland central nuclei with fine chromatin and small nucleoli. The surface cells were positive for cytokeratin 7 and TTF-1, while the round stromal cells were positive for only TTF-1 (Figures 6C, 6D). The lesional cells of the right lower lobe nodule were negative for S100, SMA, CK7, CK20, and Synaptophysin. The morphology and the immunohistochemical profile together supported the diagnosis of sclerosing pneumocytoma. All resection margins were uninvolved by the lesional cells. The excised hilar and mediastinal lymph nodes demonstrated extensive non-necrotizing granulomatous inflammation without evidence of malignancy or infection. Special stains for fungal and mycobacterial organisms were negative and based on the distribution this was most consistent with a diagnosis of sarcoidosis.

The patient has been followed for two years with stable lymphadenopathy and has remained asymptomatic.

## Discussion

The combination of a patient with both sclerosing pneumocytoma and pulmonary sarcoidosis is rare and has only been reported twice in the literature [14,15]. Most patients with pneumocytomas are asymptomatic and incidentally found to have a solid pulmonary nodule with imaging. Our patient presented with flu-like symptoms, weight loss, and fever and was found to have a concerning nodule on her right lung. Typically, sarcoidosis diagnosis requires three elements: compatible clinical and radiological manifestations, exclusion of other similarly presenting diseases, and histopathological diagnosis of noncaseating granulomas [16]. As our patient had an isolated lung solid lung nodule and marked lymphadenopathy, this presentation was concerning for malignancy.

Importantly, clinicians should be aware that fine needle aspirations and frozen sections of pneumocytomas have often been misdiagnosed as lung adenocarcinoma [17]. The associated mediastinal lymphadenopathy in the reported case appeared to represent a locally advanced malignant process which would have precluded any surgical intervention and thus an opportunity for further surgical pathology specimens; treatment would have consisted of concurrent chemotherapy and radiation. This illustrates the importance of maintaining pneumocytomas on the differential and recognizing their unique cytological features.

Despite being benign tumors, pneumocytomas can show progressive growth and can display patterns similar to those of active pulmonary tuberculosis on CT imaging [18,19]. Therefore, along with excluding a primary lung malignancy, it is critical to investigate for and rule out tuberculosis. Reassuringly, the samples of the large primary lung lesion had Gomori's methenamine silver (GMS) and Periodic Acid-Schiff with diastase (PAS-D) histochemical stains which were negative for fungal organisms as well as Ziehl-Neelsen (ZN) histochemical stain which were negative for acid-fast organisms.

Pneumocytomas are difficult to diagnose on simple FNA and often require Immunohistochemistry staining. They have been shown to consist of moderately cellular, round epithelioid cells with clear cell features, columnar cells, and spindle cells. A papillary arrangement with prominent hyalinized fibrovascular cores is the most common architectural pattern, followed by flat sheets and acinar formations. Tumor cells demonstrate mild, focally moderate nuclear pleomorphism with prominent nucleoli, hyperchromasia, nuclear elongation, nuclear overlap, and occasional nuclear inclusions and grooves. In a study by Maleki et al., the background of samples consisted of foamy macrophages in nine cases, hemosiderin pigment in six cases, and lymphoid aggregates in three cases with no mitoses and/or necrosis [20]. The staining relies on the presence of a two-cell population; TTF-1 positive and cytokeratin negative round cells as well as TTF-1 positive and cytokeratin positive surface cells. Pneumocytomas have two types of tumor cells, which can be histologically recognized as surface cuboidal cells and stroma round or polygonal cells. Most pneumocytomas have at least three of four primary histological patterns, including solid, papillary, sclerotic, and hemorrhagic regions, which vary in their proportions [21,22]. TTF-1 expression has been observed in both the surface lining cells and the pale polygonal cells. Surface lining cells are epithelial membrane antigen (EMA) positive, cytokeratin positive, and surfactant apoprotein A positive, whereas the polygonal cells are EMA positive, cytokeratin negative, and surfactant apoprotein A negative. The neuro-endocrine markers synaptophysin and chromogranin are typically negative [2].

## Conclusions

Pneumocytomas are extremely rare benign lung tumors that often mimic lung malignancy in appearance and are occasionally mistaken for adenocarcinoma on biopsy. While pneumocytomas have rarely been reported to resemble active pulmonary tuberculosis, there does not appear to be an association with any other granulomatosis disease, with this only being the third reported case with sarcoidosis. While there does not appear to be any relationship between the two entities, this is an interesting case of a patient with two rare lung pathologies.
